# A policy analysis of sleep-related legislation for Canadian licensed childcare facilities

**DOI:** 10.1186/s12889-024-20150-3

**Published:** 2024-09-27

**Authors:** Wendy A. Hall, Melissa Moynihan, Graham J. Reid, Robin McMillan

**Affiliations:** 1 University of British Columbia School of Nursing, Vancouver, BC Canada; 2 British Columbia Child Health Research Institute, Vancouver, BC Canada; 3 Department of Psychology, The University of Western Ontario, London, ON Canada; 4 Department of Family Medicine, Schulich School of Medicine and Dentistry, The University of Western Ontario, London, ON Canada; 5 Department of Paediatrics, Schulich School of Medicine and Dentistry, The University of Western Ontario, London, ON Canada; 6 Canadian Child Care Federation, Ottawa, ON Canada

**Keywords:** Sleep, Naps, Childcare, Canadian, Policy

## Abstract

**Background:**

National legislative guidelines for sleep and rest are lacking in the Canadian licensed childcare sector. No review of Canadian legislation for licensed childcare facilities has focused on sleep. This paper provides a review of the Canadian provincial and territorial legislative landscape, regarding sleep, rest, and naps in licensed childcare centers.

**Methods:**

Childcare statutes and regulations for each province and territory were identified and downloaded on a particular date. Statutes and regulations were reviewed focusing on sections articulating licensed childcare facility mandates governing sleep, rest, naps, and sleep equipment. An excel file was used to facilitate systematic data retrieval and comparisons across provinces and territories. Two authors developed and discussed themes that summarized data from the documents.

**Results:**

No statutes indicated recommendations for sleep, rest, or naps. Only one regulation defined rest (Alberta). Our analysis of regulations identified four themes representing sleep, rest, and naps: programming (general programming, daily programming); space (dedicated space, amount of space, age-specific space); equipment (developmental appropriateness, acceptable sleep equipment, age-specific equipment); and safety (staffing during sleep/rest, sleep position, sleep monitoring, sleep equipment safety, prohibited practices). In Canada, minimal regulatory consistency is evident in required sleep programming, space, acceptability of sleep equipment, and sleep safety considerations. Most jurisdictions’ regulations indicated necessity for developmentally appropriate rest or sleep areas and equipment, in particular for infants, but there was minimal consistency in defining infant age groups.

**Conclusions:**

Although we identified themes related to sleep across regulations, childcare regulations differ in their definitions of infants and specifications for children’s sleep and rest in licensed Canadian childcare facilities. Without adequate definitions in legislative components of appropriate sleep duration linked to children’s developmental stages, childcare facilities lack guidance to support healthy sleep for children in their care. Future research can examine translation of healthy sleep guidelines into government legislation and mandates for sleep, rest, and naps among young children in licensed childcare.

## Background

Sleep is fundamental to health and quality of life for children [1]. Healthy sleep is usually defined by adequate duration, appropriate timing, good quality, and the lack of sleep disturbances/disorders [2]. Sleep-wake regulation and sleep states evolve rapidly during the first year of life, with continued maturation across childhood. Twenty-four hour sleep duration in young children includes developmentally-appropriate daytime naps and nighttime sleep [3]. Care providers require information about age-appropriate night-time sleep duration, but also for the frequency and duration of naps in early development [3]. A recent longitudinal study of French children reported that, while mean nighttime sleep duration remained relatively stable from 1 to 5.5 years of age, mean daytime sleep decreased from 2 h to 58 min at age 1, to 2 h at age 2, and 1.5 h at age 3.5 [4].

In 2019, the World Health Organization published physical activity, sedentary behaviour, and sleep guidelines for children under 5 years of age [5]. The guidelines indicated that, for the greatest health benefits, children under 5 should meet all the recommendations for physical activity, sedentary behaviour, and sleep in a 24-hour period. Canadian 24-hour movement guidelines for children (0 to 4 years) integrate physical activity, sedentary behaviour, and sleep [6]. They report high quality evidence and strong recommendations for good quality sleep that includes naps for infants less than a year and 1- to 2-year-old children, and the possibility of naps in 3- to 4-year-old children [6].

Studies have demonstrated wide variation in parent-reported sleep duration for North American children. A recent U.S. Centers for Disease Control and Prevention report, based on parent-reported sleep duration, indicated that short sleep duration affected 40.3% of infants aged 4–11 months, 33.3% of children aged 1 to 2, and 34.8% of children aged 3 to 4 [7]. A Canadian study found that, based on parent-reported 24-hour sleep duration, 83.9% of 3- to 4-year-old children met sleep duration recommendations, specifically 10–13 h [8]. Sleep health is a public health issue in Canada and elsewhere [9]. Inadequate sleep affects children’s development, emotional regulation, quality of life, and mental and physical health [10]. Horváth and Plunkett, in a review of effects of napping on children’s cognitive functioning, argued that, although evidence has been conflicting, for memory consolidation and generalization, the weight of evidence indicates a beneficial effect of napping for children under 5 years of age [11].

For young children, we need to consider sleep in childcare settings. A recent environmental scan of Canadian childcare resources targeting improvements in health behaviours for children 5 years of age or younger reported only one source devoted to sleep (government-developed information sheet) and recommended resource development for sleep [12]. The authors were unable to locate policy studies about children’s sleep and naps in licensed childcare settings. Moon and colleagues examined U.S. state regulations for infant sleep positioning (back to sleep), crib safety, and bedding safety [13] and Staton and colleagues examined observed compliance with infant safe sleeping guidelines in a stratified sample of 18 Queensland licensed childcare services [14]. Other studies have examined Canadian provincial and territorial policies regarding the regulation of multi-age groupings in licensed centre-based child care, which included consideration of staff/child ratios, space, and sleep conditions [15].

Attention to children’s sleep/rest/naps in childcare facilities is important given data from a Statistics Canada survey on childcare arrangements across Canada [16]. In 2023, 56% of Canadian children aged 0 to 5 years were in childcare and 26% of children who were not in childcare were on a waitlist [16]. Despite significant numbers of children experiencing childcare, the nature of Canadian legislation about sleep in licensed childcare facilities has not been studied. Legislation governs licensed childcare facilities in Canada. Statutes are laws/acts; regulations describe the application and enforcement of acts [17]. Manuals are documents supported by government departments and intended to help childcare providers enact regulations by interpreting and supplementing the information available in regulations; they do not have the force of law.

### Aim

This paper aimed to review and analyze the Canadian provincial and territorial statutes and regulations regarding sleep, rest, and naps in licensed Canadian childcare centers.

### Methods

Between March and November 2023, we reviewed Canadian provincial and territorial statutes and regulations to inform our policy analysis of sleep in licensed daycares. We used the approach outlined by Miljand, who indicated that policy reviews can take the form of conducting a content analysis where documents are systematically categorized [18]. Frequencies for identified categories are calculated. A narrative summary of the evidence base is created, which involves description of main document characteristics, with results summarized, often in table format [18]. We undertook a multi-context review, by looking across geopolitical barriers (provinces and territories) and revealing variation in statutes and regulations about how sleep/rest/naps in licensed childcare facilities is conceptualized, and in a few cases, operationalized. We departed from Miljand’s methods by examining provincial and territorial statutes and regulations as opposed to published studies. We describe our search activities and exhaustively describe our inductive content analysis.

### Data collection

Multiple strategies were used to identify relevant policy documents. First, in March and April, 2023, preliminary internet searches were independently conducted by two authors. The search terms “childcare regulations by province and territory” and “provincial and territorial regulations for childcare and sleep”, were used by the first author. The second author used the terms “child care licensing regulations” for each province and territory (e.g., “British Columbia child care licensing regulations”). During that process, we found statutes and regulations, as well as licensing manuals, frameworks, guidelines, and handbooks.

Second, the university government documents librarian was consulted and recommended using the Canadian Legal Information Institute (CanLII) to identify legal documents. CanLII is a non-profit organization that provides open online access to legislative documents. In May, 2023, CanLII was used to locate and download all of the statutes and regulations for the 10 provinces and 3 territories. Our summary of the statutes, regulations, and licensing manuals was reviewed with the librarian who made no recommendations for additional materials. Third, a university law reference librarian was consulted but did not identify additional relevant materials.

We created folders on a shared drive that included the Child Care Statutes and Regulations that we downloaded from the CANLII website on July 4th, 2023 for each province and territory. Because the legal documents were constantly undergoing minor changes, we wanted to date the timing for all of the statutes and regulations we used for analysis. The Child Care Regulation for the Yukon was not accessible from the website on July 4, but was downloaded on July 11 after technical issues had been resolved. A summary of the statutes, regulations, and manuals was developed for each jurisdiction. Because our analytic focus was on statutes and regulations, they are summarized in Table 1.

**Table 1:** Provincial and Territorial statutes and regulations

Jurisdiction	Statutes* and Regulations*
Alberta	Early Learning and Child Care Act, SA 2007, c E-0.1, https://canlii.ca/t/55pzc Child Care Licensing Regulation, Alta Reg 143/2008, https://canlii.ca/t/54wkf
British Columbia	Community Care and Assisted Living Act, SBC 2002, c 75, https://canlii.ca/t/549r3 Child Care Licensing Regulation, BC Reg 332/2007, https://canlii.ca/t/560wd
Manitoba	The Community Child Care Standards Act, CCSM c C158, https://canlii.ca/t/55zcz Child Care Regulation, Man Reg 62/86, https://canlii.ca/t/55zjw
New Brunswick	Early Childhood Services Act, SNB 2010, c E-0.5, https://canlii.ca/t/55h4q Licensing, NB Reg 2018-11, https://canlii.ca/t/5617s
Newfoundland & Labrador	Child Care Act, SNL 2014, c C-11.01, https://canlii.ca/t/53mvh Child Care Regulations, NLR 39/17, https://canlii.ca/t/55hfh
Northwest Territories	Child Day Care Act, RSNWT 1988, c C-5, https://canlii.ca/t/560th Child Day Care Standards Regulations, RRNWT 1990, c C-3, https://canlii.ca/t/54w7r
Nova Scotia	Early Learning and Child Care Act, RSNS 1989, c 120, https://canlii.ca/t/54x0f Day Care Regulations, NS Reg 193/2010, https://canlii.ca/t/54txf
Nunavut	Child Day Care Act, RSNWT (Nu) 1988, c C-5, https://canlii.ca/t/549mj Child Day Care Standards Regulations, RRNWT (Nu) 1990, c C-3, https://canlii.ca/t/kh98
Ontario	Child Care and Early Years Act, 2014, SO 2014, c 11, Sch 1, https://canlii.ca/t/556h3 General, O Reg 137/15, https://canlii.ca/t/55qmh
Prince Edward Island	Early Learning and Child Care Act, RSPEI 1988, c E-0.1, https://canlii.ca/t/53mzq Early Learning and Child Care Act Regulations, PEI Reg EC819/16, https://canlii.ca/t/53glk
Quebec	Educational Childcare Act, CQLR c S-4.1.1, https://canlii.ca/t/55ptl Educational Childcare Regulation, CQLR c S-4.1.1, r 2, https://canlii.ca/t/561cd
Saskatchewan	The Child Care Act, SS 2014, c C-7.31, https://canlii.ca/t/560vw The Child Care Regulations, 2015, RRS c C-7.31 Reg 1, https://canlii.ca/t/554sj
Yukon	Child Care Act, RSY 2002, c 30, https://canlii.ca/t/55p6r Child Care Centre Program Regulation, YOIC 1995/87, https://canlii.ca/t/52s1d

*Statutes and regulations were accessed from CanLII website on July 4, 2023, with the exception of the regulation for the Yukon which was accessed from CanLII on July 11, 2023

We worked with the Canadian Child Care Federation (CCCF), which is a partner in this policy review. They provided staff resources to connect the investigators to affiliates (legally constituted provincial/territorial organizations involved in Early Learning and Child Care service delivery to children and families). Those groups have first-hand experience with legislative materials. In discussion with the CCCF, we developed a document briefly listing the information we had located on statutes, regulations, and licensing manuals to send to provincial and/or territorial CCCF affiliates. On July 11, the email developed by two of the authors and the documents were shared with the CCCF.

A total of 12 CCCF affiliates and 11 affiliate contacts received the email and 5 responded. Affiliates in Manitoba and Saskatchewan confirmed that we had all of the necessary documents. The Alberta affiliate sent us a link to a second related manual: The Family Day Home Standards. The Ontario affiliate sent us the link to the “Joint Statement on Safe Sleep: Preventing Sudden Infant Deaths in Canada”, published by the Public Health Agency of Canada [19], which is only identified in the Ontarian regulations. The Prince Edward Island affiliate sent us a link to the Prince Edward Island Early Learning Framework and to an email discussion about potential changes to regulations for group size for infant nap rooms.

### Analysis

The first and second authors read the statutes and regulations (defined in the background section) and identified and abstracted the text for all references to sleep, rest, and naps in the statutes and regulations. Summary files were created for documents from each jurisdiction that included references to sleep, naps, and rest.

Because none of the statutes made any mention of sleep, rest, or naps, only the regulations provided text for coding. The first author developed word documents identifying themes for sleep, rest, and naps for regulations for each jurisdiction. The second author independently created summary tables of the regulations referring to sleep, rest, and naps for the document analysis. Using an iterative process, the first author combined information from the summary tables to enhance the themes and subthemes in the word documents. Over 3 months, those authors reached consensus on themes and subthemes and the final structure of the findings.

In the course of our analysis, we identified regulatory statements that were irrelevant to daytime sleep/rest/naps for healthy children, so we did not incorporate them in our codes. For example, in the British Columbia regulations, there was a statement about child illness and providing a quiet and clean resting area for the child. A similar statement was made in the Newfoundland and Labrador regulations.

During our analysis, we noticed that jurisdictional regulations referenced a variety of sleep equipment in different contexts. In some, it was in sections about safety. In others, it was in sections describing areas for particular activities and facility requirements. As a result, we conducted another search of the regulations for the terms: crib, cot, mat, cradle, bassinette, bed, bunk bed, and playpen.

## Results

The statutes stated broad aims which varied in their level of detail. There were references to the health and wellbeing of children; but sleep was never mentioned. For example, the Ontario statute states: “The purposes of this Act are to foster the learning, development, health and well-being of children and to enhance their safety.” The Québec statute indicated the province’s aim to enhance the quality of the educational services for children before their admission to school, to ensure the health and safety of the children receiving childcare services, particularly those with special needs or who live in precarious socio-economic situations, foster their development, educational success and well-being and provide them with equality of opportunity. Because sleep/rest/naps were omitted from statutes, our analysis focused on regulations.

The regulations provided references to sleep, rest, and naps. Only one regulation defined rest (Alberta). Our analysis of regulations identified four themes representing sleep, rest, and naps: programming (general and daily); space (dedicated, amount, age-specific); equipment (developmentally appropriate, acceptable, age-specific); and safety (staffing, positioning, monitoring, equipment, prohibited practices) (See Table 2).

**Table 2:** Themes and subthemes in Canadian provincial and territorial childcare regulations related to sleep, rest, and naps

		Themes and Subthemes^2^			
Province/ Territory	Regulation (Current to date^1^)	Programming	Space	Equipment	Safety
Alberta	Child Care Licensing Regulation, Alta Reg 143/2008 (2023-06-27)	None	None	• Acceptable sleep equipment • Age-specific	• Staffing during sleep/rest • Sleep equipment safety • Prohibited practices
British Columbia	Child Care Licensing Regulation, BC Reg 332/2007 (2023-06-20)	None	• Dedicated • Age-specific	• Developmental appropriateness	• Sleep position • Sleep equipment safety • Prohibited practices
Manitoba	Child Care Regulation, Man Reg 62/86 (2023-06-27)	• General	• Dedicated • Amount • Age-specific	• Developmental appropriateness • Age-specific	• Sleep equipment safety
New Brunswick	Licensing, NB Reg 2018-11 (2023-06-15)	• Daily	• Dedicated • Amount • Age-specific	• Age-specific	• Sleep equipment safety • Prohibited practices
Newfoundland & Labrador	Child Care Regulations, NLR 39/17 (2022-09-01)	• General • Daily	• Amount • Age-specific	None	• Sleep monitoring • Sleep equipment safety • Prohibited practices
Northwest Territories	Child Day Care Standards Regulation, RRNWT 1990 c C-3 (2023-06-30)	• General • Daily	• Dedicated • Age-specific	• Acceptable sleep equipment	• Staffing during sleep/rest • Sleep equipment safety
Nova Scotia	Day Care Regulations, NS Reg 193/2010 (2023-06-30)	• General • Daily	• Age-specific	• Age-specific	• Staffing during sleep/rest • Sleep equipment safety • Prohibited practices
Nunavut	Child Day Care Standards Regulations, RRNWT (Nu) 1990 c C-3 (2023-06-23)	None	• Dedicated • Age-specific	• Acceptable sleep equipment	• Staffing during sleep/rest • Sleep equipment safety
Ontario	General, O Reg 137/15 (2019-12-08)	• General • Daily	• Dedicated • Age-specific	• Age-specific	• Staffing during sleep/rest • Sleep position • Sleep monitoring • Prohibited practices
Prince Edward Island	Early Learning and Child Care Act Regulations, PEI Reg EC819/16 (2023-06-19)	• General	• Age-specific	• Age-specific	• Sleep equipment safety • Prohibited practices
Québec	Educational Childcare Regulation, CQLR c S-4.1.1, r 2 (2023-01-01)	None	• Dedicated • Age-specific	• Age-specific	• Sleep equipment safety • Prohibited practices
Saskatchewan	The Child Care Regulations, 2015, RRS c C-7.31 Reg 1 (2023-06-26)	None	• Dedicated • Age-specific	• Developmental appropriateness	• Staffing during sleep/rest
Yukon	Child Care Centre Program Regulation, YOIC 1995/87 (2020-12-31)	• General	• Dedicated • Amount	• Acceptable sleep equipment	• Sleep equipment safety

^1^Current to date refers to dates in brackets.^2^Bullet points under each theme are sub-themes. Specifically, Programming sub-themes: (1) General programming, (2) Daily programming; Space sub-themes: (1) Dedicated space, (2) Amount of space, (3) Age-specific space; Equipment sub-themes: (1) Developmental appropriateness, (2) Acceptable sleep equipment, (3) Age-specific sleep equipment; Safety sub-themes: Staffing during sleep/rest, 2. Sleep position, 3. Sleep monitoring, (4) Sleep equipment safety, (5) Prohibited practices

The themes and subthemes (see Table 3) were predicated on the regulations including the differences in how regulatory statements handled programming, space, equipment, and safety.

**Table 3:** Qualitative themes and subthemes for sleep, rest, and naps (number of jurisdictions)

Programming	Space	Equipment	Safety
• General aims (5) • Opportunities for sleep/rest/or naps	• Dedicated sleep space in centre (8) • Separate from play area • Quiet space	• Acceptable sleep equipment (5) • Complies with legislation • Types	• Infant Sleep position (2)
• Daily activities (3) • Amount of daily sleep/rest • Timing of daily sleep/rest	• Nature of sleep space (4) • Amount of floor area • Space between sleeping equipment	• Developmentally appropriate for size and age (3)	• Sleep equipment safety (10) • Cleanliness • Complies with legislation
	• Age-specific (10) • Numbers • Separate from play/activity space	• Age-specific (7) • Infant • Child	• Sleep monitoring (2) • Visual checks • Audio monitor
			• Staffing during sleep/rest (6) • Staff ratio • Maximum group size
			• Prohibited practices (8) • Sleep deprivation • Beverages/food • Inappropriate sleep equipment

Themes and Sub-themes across regulations.

**1) The programming** theme consists of two sub-themes. The first is general programming. The general aims for Ontario, Nova Scotia, Prince Edward Island, Newfoundland and Labrador, Northwest Territories, and Yukon refer to incorporating time for rest, quiet play or time, and/or sleep into the day. Manitoba, Ontario, Newfoundland and Labrador, North West Territories, and Yukon regulations indicate facilities should take into consideration the age/needs of the child for sleep/rest. Regulations for Nova Scotia and Yukon state that rest periods must be included in the day for children. Regulations for the remaining jurisdictions provide no general programming aims for sleep or rest (British Columbia, Alberta, Saskatchewan, Québec, New Brunswick, and Nunavut).

The second sub-theme is daily programming. Three jurisdictions specify regulations for either amount of sleep or rest (Ontario, New Brunswick) or timing of sleep or rest (Northwest Territories). Ontario regulations require written policies and procedures describing sleep opportunities and child behaviours that are communicated to parents on a daily basis. Ontario, Newfoundland and Labrador, and Nova Scotia regulations require facility-based written records, including children’s daily amounts and timing of sleep.

2) The **space theme** has three subthemes: dedicated space, amount of space, and age-specific requirements. All jurisdictions, except Alberta, Newfoundland and Labrador, Nova Scotia, and Prince Edward Island, indicate dedicated sleeping space is required for children. Manitoba’s and Yukon’s regulations express space requirements for children in general. Rest space, rather than sleep, is regulated in some jurisdictions: Québec’s regulations incorporate a ‘rest room’ in the ‘play area’ and New Brunswick’s regulations refer to rest areas.

Amount of space devoted to sleep varies by jurisdiction. Four jurisdictions, Manitoba, New Brunswick, Newfoundland and Labrador, and the Yukon, specify amount of dedicated space for sleep. Manitoba states amount of space required per child for sleeping; New Brunswick and Newfoundland and Labrador specify amount of space required between sleep equipment. Yukon’s regulations acknowledge that the required amount of space per child includes space for sleep.

All but two regulations (Alberta and the Yukon) identify infant-based age-specific sleep spaces but there is significant variation in how jurisdictions define infants. Three jurisdictions specify that infant sleep spaces have to be separate from other activities (Ontario, Prince Edward Island, Newfoundland and Labrador). Some sleeping/resting space requirements for infants are expressed as square metres of space (Saskatchewan) or amount of space between sleep equipment for infants (New Brunswick, Nova Scotia). Québec regulations indicate that children under 18 months must be provided with a minimum net area of 4 m² per child with one of two rooms for resting. Regulations also stipulate that younger children require a separate quiet sleeping space: Nunavut and Northwest Territories (18 months); and British Columbia (36 months).

3) The **sleep equipment** theme is comprised of three subthemes: developmental appropriateness, acceptability, and age-specific requirements. British Columbia’s and Saskatchewan’s regulations only specify the necessity for developmentally/age appropriate sleep equipment. Manitoba regulations refer to sleep equipment that is suitable for the developmental capabilities of the children. While we grouped this with developmental appropriateness, it should be acknowledged that using the term children’s developmental capabilities implies a focus distinct from age. The Northwest Territories and Nunavut regulations indicate that each child who rests or sleeps must be provided with a cot, bed, or sleeping mat. For Alberta, regulations indicate that mats, beds, cribs, cradles, or bassinets are acceptable for sleep or rest. The Yukon regulations only indicate that equipment (it specifies crib, cots, beds or mats) must be suitable for child size and age. Newfoundland and Labrador had no regulations under the equipment theme. Only the Northwest Territory’s regulations indicate that traditional Aboriginal sleep equipment is acceptable.

Using age criteria, acceptable sleep equipment (e.g., cots, cribs, mats, bedding) is identified by regulations for Alberta, Manitoba, Ontario, Québec, New Brunswick, Prince Edward Island, and Nova Scotia. For example, the Alberta regulations indicate that each infant receiving care is provided with a separate crib, cradle or bassinet. New Brunswick’s regulations require a crib or portable playpen for each child under 15 months and specify a cot or nap mat appropriate for a child’s height for each child who is at least 15 months of age and under 5 years. Manitoba’s and Québec’s regulations indicate the necessity for a separate playpen or crib for each child less than 18 months of age. Ontario’s regulations require a crib or a cradle for each child who is younger than 12 months. The regulations for Prince Edward Island indicate a crib or alternative bed is necessary for children under 12 months of age. Nova Scotia refers to specific equipment for infants, toddlers, and preschoolers.

As can be observed from the subthemes that specified age-related sleep space and sleep equipment, jurisdictions provided no or inconsistent definitions of infants by age. The regulations for British Columbia, New Brunswick, Prince Edward Island, Québec, the Northwest Territories, Nunavut, and Yukon do not explicitly define ages for infants or toddlers, although Québec requires specific arrangements for children under 18 months of age. The regulations for Nova Scotia, Ontario, and Saskatchewan define infants as less than 18 months of age while Alberta’s regulations define infants as under 19 months. Manitoba’s and Newfoundland and Labrador’s regulations define infants as less than 2 years of age.

4) The **safety theme** has five subthemes: staffing during sleep/rest, sleep position, sleep monitoring, sleep equipment safety, and prohibited practices. Required staffing during sleep/rest, by age group, is specified by regulations from Alberta. Ontario’s regulations indicate that the infant age group can never have a reduced staff ratio. Regulations for four other jurisdictions only indicate that maximum group sizes do not apply for children who are resting (Saskatchewan, Nova Scotia, Northwest Territories, Nunavut). Other jurisdictions do not specify explicit staffing requirements during sleep, rest, or naps.

Sleep positioning (i.e., back to sleep for infants) is stipulated by regulations in just two jurisdictions (British Columbia, Ontario). Two jurisdictional regulations specify sleep monitoring, specifically direct visual checks of children and/or using sleep monitors to observe children (Ontario, Newfoundland and Labrador).

For sleep equipment safety, cleanliness of equipment is specified in 9 of 13 jurisdictional regulations (British Columbia, Manitoba, New Brunswick, Newfoundland and Labrador, Northwest Territories, Nova Scotia, Yukon, Nunavut, and Québec). For five provinces (Alberta, Manitoba, New Brunswick, Prince Edward Island, and Québec) regulations state that sleep equipment must comply with the Canadian Consumer Safety Product Act [20]. The Prince Edward Island and New Brunswick regulations specify compliance for children under 12 and 15 months of age respectively. Alberta regulations indicate that for infants (no specification for age) cribs, cradles, or bassinets are used in accordance with the Consumer Product Safety Act. In contrast, Manitoba regulations specify that sleep equipment for each child in a facility must comply with the Consumer Product Safety Act. In Québec, regulations refer only to cribs and playpens having posts and slats that comply with the Consumer Safety Product Act. Yukon Territory regulations refer to cots, cribs, beds, or mats meeting the requirements of the Hazardous Products Act (Canada) for each child [21]. Nova Scotia regulations state that cribs or portable cribs provided for infants meet the Federal or Provincial legislation respecting cribs, cradles, and bassinets. Northwest Territory and Nunavut regulations specify that any sleep equipment meets the requirements of the Fire Marshal. Regulations for British Columbia, Saskatchewan, Ontario, and Newfoundland and Labrador do not specify safety considerations for sleep equipment.

The regulations include some prohibited practices. Five sets of provincial regulations indicate bans on supplying sustenance (beverages and food) while children are napping/resting (Alberta, New Brunswick, Nova Scotia, Prince Edward Island, Newfoundland and Labrador). Regulations for Ontario, British Columbia, and Newfoundland and Labrador prohibit depriving a child of basic needs for sleep/rest. Québec regulations stipulate that children should not be confined to playpens or beds when not sleeping and prohibit the use of bunk beds, bassinettes, or cradles as sleep equipment. Nova Scotia and Newfoundland and Labrador prohibit playpens.

## Discussion

Our policy review has highlighted a number of problems with the Canadian provincial and territorial legislative landscape, regarding sleep, rest, and naps in licensed Canadian childcare centers. Most Canadian jurisdiction statutes make broad aspirational aims about children’s health and wellbeing; however, promoting children’s healthy sleep has been entirely overlooked. This finding fits with Carson and colleagues’ environmental scan of existing Canadian childcare resources where only one resource targeted sleep health behavior for children under 5 years of age [12].

It is concerning that regulations from only two jurisdictions (Ontario, British Columbia) incorporate sleep positioning (back to sleep) and two (Ontario, Newfoundland and Labrador) include sleep monitoring. No assumptions can be made about child care providers and administrators promoting sleep safety without supportive government policies and access to sleep education. The Canadian Pediatric Society has provided clear guidelines about how to create safe sleep environments for babies [22]. In situ observations conducted in 18 Australian childcare facilities revealed that 83% of those facilities were non-compliant on at least one national safe sleep guideline, including prone or side positioning and loose and soft materials in cots [14]. Engaging Canadian organizations supporting children’s health and the Canadian Sleep Society to lobby politicians could bring about legislative changes to enhance children’s sleep safety in licensed childcare facilities.

Because most regulatory guidelines for sleep space and equipment are specific to children’s ages and there are few regulatory directives for sleep timing and amount for children, the statutes and regulations present considerable gaps in guidance for childcare providers. Recent work has emphasized the importance of regularity of sleep timing (going to sleep and waking) for children’s sleep health [3]. Just two provinces (Ontario, New Brunswick) have regulations that specify a daily rest period not exceeding 2 h in length for toddlers/preschoolers and, in the second instance, infants and preschoolers. Because sleep duration needs are constantly changing between the ages of 4 months and 18 years [3], providing regulatory guidance about appropriate sleep/rest periods for children’s developmental stages potentially avoids inappropriate mandates by childcare facilities for children’s sleep.

The aspirational assertions in some statutes refer to equitable treatment of children. For example, the Québec statute specifically emphasizes the health and safety of the children who live in precarious socio-economic situations; the Ontario statute refers to respecting equity, inclusiveness, and diversity in communities; and the New Brunswick statute refers to creating inclusive environments that respect the diversity related to race, colour, religion, place of origin, disability, family status, gender identity, and social condition. Lack of attention to sleep and rest in statutes and regulations for licensed childcare facilities is particularly problematic because studies have associated children’s lack of healthy sleep with experience of suboptimal socio-demographic factors. Pre-school aged children (aged 3 to 5 years) living in environments that were inadequate for night-time sleep, (e.g., increased noise, cold, light), had shorter nocturnal sleep duration [23]. Williamson et al. reported that increased cumulative socio-demographic risk factors (e.g., single caregiver, a crowded home) are predictive of toddlers’ and preschool children’s (2 to 5 years of age) insufficient sleep and at least one symptom of pediatric insomnia [24]. Jones and Ball reported that three-year old children with low socio-economic status were less likely to be exposed to sleep hygiene at bedtime, had shorter night-time sleep, and longer day-time naps [25]. These findings suggest greater need and benefit from naps for disadvantaged groups. Lack of guidelines in statutes and regulations for promoting children’s age-appropriate sleep duration and healthy sleep practices highlights government instrumentalities’ failure to recognize critical contributions of sleep to children’s wellbeing and indicates ‘lip service’ to inclusivity and equity.

The American Academy of Sleep Medicine consensus statement links sleep duration within the recommended range for age to optimal physical and mental health outcomes (e.g., improved learning, memory, emotion regulation, and quality of life) and links inappropriate sleep duration to hypertension, diabetes, and obesity [10]. For example, Deng et al. found that short sleep duration was significantly associated with obesity in toddlers (1–2 years) and preschool-aged (3–5 years) children [26]. Overweight/obesity is a major concern for Canadian children [27]. A review of primary care electronic records indicated that 6% of Ontarian children aged 0 to 5 years were categorized as overweight/obese [27]. Because the statutes and regulations do not provide any specificity about recommendations for healthy sleep, many Canadian children in childcare environments may not be receiving adequate amounts of daytime sleep, which could put them at risk for overweight/obesity. Jurisdictional regulatory guidelines can optimize daytime sleep, with positive effects for the one-third of Canadian children who are in childcare.

There are major differences in recommendations for children’s napping. Canadian 24-hour movement guidelines for children (0 to 4 years) indicate high quality evidence and strong recommendations for good quality sleep that includes naps [6]. Specifically, naps for all infants less than a year and 1- to 2-year-old children, and the possibility of naps in 3- to 4-year-old children [6]. On the other hand, a systematic review by Thorpe and colleagues concluded that, beyond age 2, napping is associated with later night sleep onset and both reduced sleep quality and duration [28]. Out of 26 studies reviewed, their review included 14 studies where children were up to 6 years of age. For example, 2 studies reporting nighttime shorter sleep duration had samples with children’s ages ranging from 42 to 72 months [29, 30] and 1 study only sampled children aged 60 months [31]. Reaching conclusions about effects of napping on night sleep quality and duration is problematic when the ages of children in the majority of studies reviewed by Thorpe [28] exceeded ages regarded as appropriate for naps in movement guidelines [6].

Staton and colleagues indicated that between 15% and 30% of preschool children (3- to 5-years of age) will not have achieved monophasic sleep and require daytime sleep, in particular, children living in situations of social disadvantage [32]. Newton et al. reported that non-white children who had ceased napping by their third birthday tended to have lower cognitive ability than non-white still napping children [33]. Staton and colleagues called into question practices in licensed childcare facilities where a particular period of sleep/rest is mandated; however, the evidence they have presented, that supports problems arising from napping in children more than 2 years of age, also suggests that naps serve an important function compensating for poor night-time sleep [32]. Some investigators hypothesize that napping has positive benefits for learning, memory and emotion regulation, but only for children who are regular nappers [34].

It is important to carefully consider potential compounding effects of difficulties with nighttime sleep at home and naps in childcare settings. A Canadian study documented 38% of 1 to 3-year-old children in licensed childcare settings had maternal report of sleep problems at home which were positively correlated with mothers’ assessments of 3-year-old children’s internalizing and withdrawn behaviours in comparison to children without sleep problems at the same age; in addition, difficulties settling for naps in their licensed childcare settings were positively associated with childcare providers’ assessments of children’s problems concentrating and higher scores on anxiety and aggression and significantly negatively correlated with peer competence [35]. Regulatory statements are missing the opportunity to improve children’s healthy sleep by omitting appropriate guidelines for daytime sleep/rest/naps.

Translating policy into practice can be challenging. Staton and colleagues found that, despite staff members indicating high levels of confidence and knowledge about complying with sleep standards, 83% of childcare settings caring for infants did not comply with Australian national legislated safe sleeping guidelines [14]. A related point is the lack of professional groups who could support childcare settings in implementing best practices for sleep. There is a dearth of training in sleep and circadian rhythms for Canadian health care professionals and allied disciplines [36]. Development and deployment of practices to optimize sleep in childcare requires a collaborative approach. Clear direction from legislated statutes and regulations for promoting healthy childhood sleep would provide a foundation. Support and direction for training of childcare providers about children’s healthy sleep would benefit from collaborations at the national (e.g., CCCF and the Canadian Sleep Society), and provincial/territorial levels.

Developing national standards for sleep, rest, and naps in licensed childcare facilities has been undertaken in other countries, e.g., Australia [37]. These standards include ensuring needs for sleep and rest are met, risk assessments for sleep equipment and bedding, back to sleep, access to fluids and food, location and arrangement of physical environments, and visibility and direct supervision of sleepers [37]. The Australian system has a history of regular government proposals of national guidelines and funding to support them. The constitution of the Canadian federation has placed responsibility for health and education with the 10 provinces and three territories, leading to a dearth of national standards. In both Australia and Canada, federal governments rely on states, territories, and provinces to apply national guidelines and enforce them. In Canada, difficulties overcoming jurisdictional differences and claims to autonomous regulatory requirements would be extensive.

### Limitations

The authors did not include analysis of statutes/regulations specific to overnight stays in licensed childcare facilities. The necessity of specifying the timing of access to statutes and regulations precludes any updates of the documents. While care was taken to identify as many resources as feasible, it is possible that the authors did not identify all relevant resources.

## Conclusion

Our pioneering analysis of sleep/rest/nap requirements in Canadian statutes/regulations has highlighted the lack of attention to promoting children’s healthy sleep and important gaps and inconsistencies in provincial and territorial practices. We agree with Carson and colleagues who argued that, because large numbers of Canadian children are in childcare, childcare centres are ideal settings to promote healthy patterns of nutrition, physical activity, sedentary behaviour, and sleep [12]. Inadequate attention to appropriate sleep/nap duration linked to children’s developmental stages leaves childcare facilities without guidance to support healthy sleep for children in their care. It is particularly problematic that there is major variation in how jurisdictions define infants in terms of age when age-specific regulations are mainly expressed for infants. The lack of consistency in legislative specifications for most aspects of children’s sleep in licensed childcare facilities across Canada is indicative of a lack of attention by government to contributions of sleep to children’s health and wellbeing. Future research studies can examine effects of translation of healthy sleep guidelines and mandates for sleep, rest, and naps in government legislation for young children in licensed childcare. Studies could also explore licensed childcare facilities’ adherence to legal specifications and use of manuals to expand childcare providers’ understanding of regulatory requirements.

## Data Availability

Data sharing is not applicable to this article as no datasets were generated or analyzed during the current study.

## Abbreviations

CCCF: Canadian Child Care Federation
CanLII: Canadian Legal Information Institute
CPEs: Centres de la petite enfance
